# Immunotherapy – Strategies for Expanding Its Role in the Treatment of All Major Tumor Sites

**DOI:** 10.7759/cureus.5938

**Published:** 2019-10-18

**Authors:** Chandan Sanghera, Rohan Sanghera

**Affiliations:** 1 Department of Medicine, Imperial College London, London, GBR; 2 School of the Biological Sciences, University of Cambridge, Cambridge, GBR

**Keywords:** immunotherapy, combination immunotherapy, immune check point inhibitors, car t-cell therapy

## Abstract

Immunotherapy is widely regarded to have the ability to transform the treatment of cancer, with immune checkpoint inhibitors already in use for cancers such as advanced melanoma and non-small cell lung cancer (NSCLC). However, despite its potential, the widespread adoption of immunotherapy for the treatment of other cancers has been largely limited. This can be partly attributed to additional immunosuppressive mechanisms in the tumor microenvironment that help promote and maintain a state of T cell exhaustion. As such, the exploration of combinatory immunotherapies is an active area of research and includes the combination of immune checkpoint inhibitors with cytotoxic therapies, cancer vaccines and monoclonal antibodies against other co-inhibitory and co-stimulatory receptors. Strategies are also being employed to improve the homing, extravasation and survival of chimeric antigen receptor (CAR)-T cells in the tumor microenvironment. Furthermore, the development of immunotherapies targeted to one or multiple neoantigens unique to a specific tumor may act to enhance anti-tumor immunity, as well as reduce immune-related adverse events (irAEs). As immunotherapy evolves to become a mainstay treatment for cancer, it is imperative that optimum treatment regimens that maximize efficacy and limit toxicity are developed. Foremost, appropriate biomarkers must be identified to help tailor combinatory immunotherapies to the individual patient and hence pave the way to a new era of personalized medicine.

## Introduction and background

The notion of harnessing the host’s immune system to eliminate cancer has been well-established for years, even though the field has only started taking off relatively recently. It stems from the knowledge that the immune system is a critical player in the prevention, as well as the development and progression of cancer. The prevention of tumorigenesis is achieved via numerous mechanisms, including protection against viral-induced tumors and suppression of tumor-promoting inflammatory environments. Arguably, the most important mechanism is that malignantly transformed cells often co-express ligands associated with DNA damage and tumor antigens, which can be recognized and targeted by the innate immune system and lymphocytes of the adaptive immune system, respectively, in a process termed immune surveillance [1]. 

The ability of the immune system to recognize antigens on malignant cells and target them for destruction forms the foundations of immunotherapy [2]. However, a distinguishing hallmark of cancer is its ability to evade immune destruction in a number of distinct ways. Notably, immune editing provides a selective pressure that gives rise to a less immunogenic population of neoplastic cells [2-3]. This is augmented by other immune-evading processes, including disruption of T cell function and signaling, defective antigen presentation, and the altered production of immune-suppressive mediators such as inhibitory cytokines and immunosuppressive cells. These processes ultimately lead to the proliferation of malignant cells, clinically manifesting as cancer [3]. 

Immunotherapy holds a lot of promise, not least because it avoids the many limitations of chemotherapy and radiotherapy - the current mainstay treatments for cancer. These limitations include systemic toxicities, lack of specificity for malignant cells, recurrence of drug-resistant tumors, and the inability to target and treat micrometastasis or subclinical disease [4]. The main forms of immunotherapy are immune checkpoint inhibitors, adoptive cell transfer, cytokine therapy, and cancer vaccines. As cancer immunotherapy effectively targets the immune system, in principle it should be able to treat a broad range of tumor types independent of the underlying histology or driver mutations. However, to date, immunotherapy has only demonstrated efficacy in a select group of cancers and usually in a minority of patients with those cancers, limiting its use as a treatment. Consequently, strategies for expanding its use is an active area of research and will be the focus of this review. 

## Review

Immune checkpoint inhibitors

Immune checkpoint inhibitors have achieved notable clinical success as a novel class of immunotherapy, particularly in patients with advanced melanoma and non-small cell lung cancer (NSCLC) [4]. The primary effector cells of the adaptive immune response to cancer are T lymphocytes, including T helper cells and cytotoxic T lymphocytes (CTLs). These cells are primed and activated through interaction of their T cell receptors (TCRs) with tumor antigens presented on major histocompatibility complexes (MHCs) by antigen-presenting cells (APCs). Immune checkpoints essentially provide the co-stimulatory or co-inhibitory signals that regulate this process. Specifically, cytotoxic lymphocyte-associated protein 4 (CTLA-4) is a co-inhibitory receptor which, through binding to CD80 (B7.1) and CD86 (B7.2) ligands expressed on tumor cells and APCs, inhibits the process of T cell priming and activation. Normally, after priming and activation, CTLs migrate to the tumor where they exert their cytotoxic activity. This process is also controlled by immune checkpoints including programmed cell death protein 1 (PD-1) receptor, a co-inhibitory receptor present on activated T cells, regulatory T (Treg) cells, B cells, and natural killer (NK) cells. The ligands of this receptor, PD-L1 and PD-L2, are also expressed by both tumor cells and APCs, and once bound lead to T cell apoptosis and exhaustion, providing protection against CTL-mediated killing. As such, these inhibitory pathways are often found to be upregulated in cancer, as one of many mechanisms to evade immune surveillance. 

**Figure 1:**
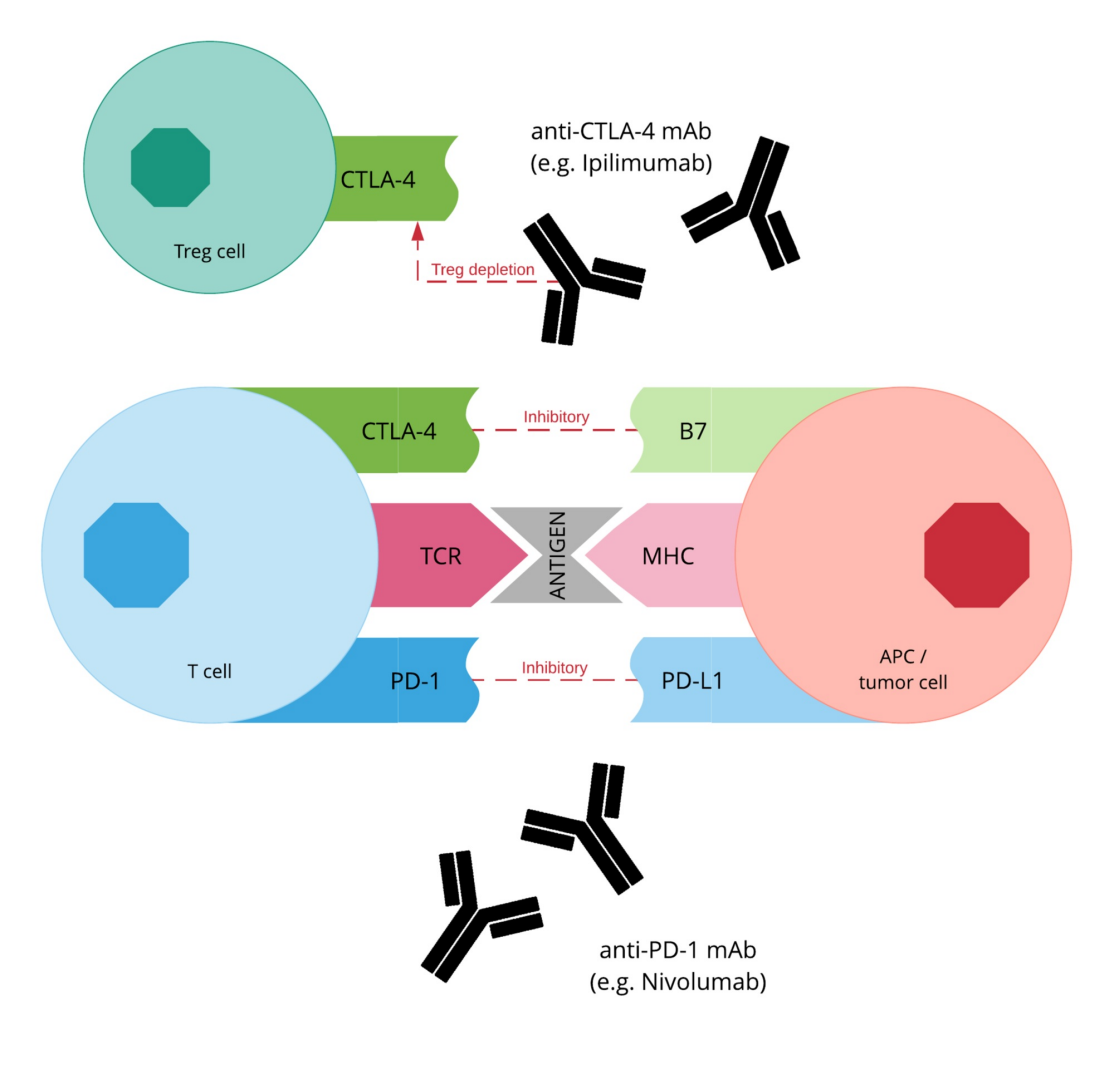
Mechanism of action of anti-CTLA-4 and anti-PD-L1 immune checkpoint inhibitors CTLA-4, cytotoxic lymphocyte-associated protein 4; MHC, major histocompatibility complex; PD-1, programmed cell death protein 1; TCR, T cell receptor; Treg cell, regulatory T cell

Given the crucial role of immune checkpoints in suppressing the anti-tumor immune response, the use of monoclonal antibodies (mAbs) as immune checkpoint inhibitors targeting CTLA-4 and PD-1/PD-L1 appears very promising, with their mechanism of action shown in Figure 1. Examples of approved immune checkpoint inhibitors include ipilimumab, an anti-CTLA-4 mAb, and nivolumab, an anti-PD-1 mAb. However, despite immune checkpoint inhibitors showing anti-tumor activity in a number of different malignancies, less than 25% of patients achieve any benefit [5]. This may in part be due to additional suppressive mechanisms that help contribute and maintain T cell exhaustion, preventing the immune system from mounting a sufficient response. For instance, targeting the PD-1 pathway alone does not result in complete restoration of T cell function and in fact expression of alternative co-inhibitory immune checkpoints has been associated with resistance to PD-1 blockade [6]. One such inhibitory receptor that is expressed on CTLs and Treg cells during exhaustion is lymphocyte activation gene 3 protein (LAG3), with efficacy studies demonstrating an enhanced response in melanoma patients treated with both nivolumab and relatlimab, an anti-LAG3 mAb [7]. Similarly, T cell immunoglobulin and mucin domain-containing 3 (TIM3) are also thought to maintain a state of T cell exhaustion and are found to be expressed on lymphocytes in a range of tumors. In fact, preclinical studies have already demonstrated a superior synergistic effect of combined TIM3 and PD-1 blockade in cancers, such as melanoma, gastric cancer, hepatocellular carcinoma (HCC), and acute myeloid leukemia (AML), which is superior to targeting either pathway alone [6,8]. 

The most studied immunotherapy combination is combined PD-1 and CTLA-4 blockade. This combination has been shown to be able to overcome T cell exhaustion and restore anti-tumor immunity in a wide range of cancers, with phase III trials demonstrating superiority of this approach in the treatment of melanoma, renal cell carcinoma, and NSCLC [9-11]. Other cancers being explored in early clinical trials include bladder cancer, ovarian cancer, colorectal cancer, breast cancer, HCC and pancreatic cancer, as shown in Table 1 [12]. This synergistic effect of dual blockade results from the alteration of different signaling pathways within T cells, with suppression of both Treg cells and inhibitory pathways within CTLs being thought of as the primary mechanism of action [12]. The action of killer inhibitory receptors (KIRs), which recognize MHC class I molecules and subsequently negatively regulate the cytotoxic activity of NK cells, is another mechanism of avoiding immune surveillance in tumor cells through retention of MHC class I expression [13]. Hence, KIR specific mAbs that are able to block this negative regulation may provide a further combinatory target. T cell exhaustion may also be overcome through tissue engineering approaches. For example, re-differentiation of pluripotent stem cells into the T cell lineage, followed by transduction with engineered TCRs or chimeric antigen receptors (CARs), has been shown to delay cancer progression in solid tumors [14]. However, it is currently unclear if this method overcomes the issue of T cell exhaustion. Interestingly, direct cell-cell contact between T cells and tumor cells has been shown to induce T cell defects after short-term incubation, suggesting that severing this contact may act to enhance T cell efficacy [15].

**Table 1:** A summary of significant combination immunotherapy regimens currently being explored in clinical trials

Trial	Tumor type	Therapy	Clinical outcome
NCT03298451	Advanced hepatocellular carcinoma	Durvalumab plus tremelimumab	Initial data from phase I/II trial suggests improved objective response rate compared to durvalumab alone.
NCT03434379	Advanced hepatocellular carcinoma	Atezolizumab plus bevacizumab	Initial data from phase Ib trial suggests improved response rate compared to monotherapy with either agent.
NCT03713593	Advanced hepatocellular carcinoma	Pembrolizumab plus lenvatinib	Initial data from phase Ib trial suggests good anti-tumor response in unresectable tumors.
NCT02425891	Triple-negative breast cancer	Atezolizumab plus paclitaxel	Improved overall survival and progression-free survival compared to monotherapy, particularly in PD-1 positive subgroup.
NCT03036488	Triple-negative breast cancer	Pembrolizumab plus chemotherapy regimen	Improved pathological complete response and event-free survival compared to chemotherapy alone.
NCT01844505	Advanced melanoma	Nivolumab plus ipilimumab	Improved overall survival compared to ipilimumab alone.
NCT02231749	Advanced renal cell carcinoma	Nivolumab plus ipilimumab	Improved overall survival and objective response rate versus sunitinib.
NCT02853331	Advanced renal cell carcinoma	Pembrolizumab plus axitinib	Improved overall survival, progression free survival and objective response rate versus sunitinib.
NCT02684006	Advanced renal cell carcinoma	Avelumab plus axitinib	Improved progression free survival compared to sunitinib, though no improvement in overall survival.
NCT02420821	Advanced renal cell carcinoma	Atezolizumab plus bevacizumab	Improved progression free survival compared to sunitinib.
NCT02985957	Metastatic prostate cancer	Nivolumab plus ipilimumab	Initial data from phase II trial suggests improved objective response rate.
NCT02039674	Advanced non-small cell lung cancer	Pembrolizumab plus carboplatin plus paclitaxel	Improved overall survival and progression free survival compared to carboplatin and paclitaxel alone.
NCT01454102	Advanced non-small cell lung cancer	Nivolumab plus ipilimumab	Improved progression free survival compared to chemotherapy treatment.
NCT02542293	Advanced non-small cell lung cancer	Durvalumab plus tremelimumab	No improvement in overall survival compared to chemotherapy.
NCT02366143	Advanced non-small cell lung cancer	Atezolizumab plus bevacizumab plus carboplatin plus paclitaxel	Improved overall survival and progression-free survival compared to bevacizumab plus carboplatin plus paclitaxel.
NCT03214250	Metastatic pancreatic cancer	Nivolumab plus gemcitabine plus paclitaxel plus APX005M	Initial data from phase Ib trial suggests promising anti-tumor response.
NCT03036098	Metastatic bladder cancer	Nivolumab plus ipilimumab	Improved overall survival and progression free survival compared to chemotherapy.
NCT02807636	Advanced or metastatic bladder cancer	Atezolizumab plus platinum-based chemotherapy	Improved progression free survival compared to atezolizumab alone.
NCT02498600	Recurrent ovarian cancer	Nivolumab plus ipilimumab	Initial data from phase II trial demonstrates improved anti-tumor response compared to nivolumab alone.
NCT02580058	Recurrent ovarian cancer	Avelumab plus doxorubicin or platinum-based chemotherapy	No improvement in overall survival or progression free survival compared to chemotherapy.
NCT02788279	Metastatic colorectal cancer	Atezolizumab plus cobimetinib	No improvement in overall survival compared to regorafenib.
NCT02060188	Metastatic colorectal cancer	Nivolumab plus ipilimumab	Initial data from phase II trial demonstrates promising objective response rate.

Another reason for the limited efficacy of immune checkpoint inhibitors in certain cancers may relate to a poor Immunoscore, which is a scoring system that classifies cancers according to the level of immune cell infiltration. The Immunoscore ranges from I0 or ‘cold’ for poorly infiltrated tumors to I4 or ‘hot’ for well-infiltrated tumors. Hot tumors have been associated with improved response rates to checkpoint blockade [16]. Hence, combinatory treatments that are able to prime the immune system prior to checkpoint inhibitor therapy are another possible approach to expand the role of immunotherapy. For example, agonistic targeting of costimulatory receptors found on T cells, such as CD137 and OX40, enhances the anti-tumor immune response by promoting T cell proliferation and survival [17-18]. Alternatively, T cells can be primed and expanded through the use of neoantigen-based vaccines or by targeting cytokines, for example, dual IL-10 and PD-1 blockade is able to enhance the function of tumor-specific CTLs [19]. Conventional treatments such as chemotherapy or radiotherapy may also have a role in improving the response to immunotherapies, for example, as neoadjuvant therapies. They are also able to upregulate the production of chemokines and cytokines, increase the expression of MHC molecules, and facilitate tumor death, enabling the release of tumor-associated antigens [12]. This, in turn, leads to enhanced antigen presentation, and T cell recruitment and activation, which results in the upregulation of inhibitory immune checkpoints. Other agents also have the potential to upregulate immune checkpoints and enhance anti-tumor immunity. Such agents include 1) vascular endothelial growth factor (VEGF) inhibitors, which are able to increase lymphocyte infiltration into tumors and reduce expression of Treg cells, 2) adenosine (P1) receptor inhibitors, which increase APC activation and reduce Treg cell expression, and 3) mitogen-activated protein kinase (MAPK) inhibitors, which promote tumor cell death and the presentation of tumor-associated antigens by enhancing MHC class I expression [12,20]. 

**Figure 2:**
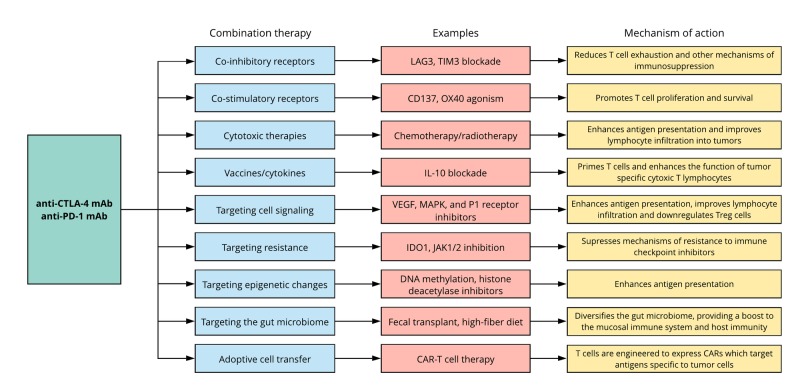
Combinatory immunotherapy approaches and their synergistic mechanisms of action CAR, chimeric antigen receptor; CTLA-4, cytotoxic lymphocyte-associated protein 4; IDO1, indoleamine 2,3-dioxygenase 1; JAK, Janus kinase; LAG3, lymphocyte activation gene 3 protein; MAPK, mitogen-activated protein kinase; PD-1, programmed cell death protein 1; TIM3, T cell immunoglobulin and mucin domain-containing 3; Treg cells, regulatory T cells; VEGF, vascular endothelial growth factor.

Another strategy to expand the use of immune checkpoint inhibitors is to target mechanisms of resistance. For example, indoleamine 2,3-dioxygenase 1 (IDO1) has been implicated in resistance to both anti-CTLA-4 and anti-PD-1 mAbs, though as of yet phase III trials have not demonstrated any clinical improvements [21]. Mutations in immune effector signaling pathways are also capable of suppressing the activity of tumor-specific T cells and provide mechanisms of resistance to treatment. For example, mutations in Janus kinase 1 (JAK1) and Janus kinase 2 (JAK2) are associated with loss of interferon-gamma (IFNγ) responsiveness and antigen presentation. This has been shown to result in resistance to PD-1 blockade, which can be overcome by inhibition of JAK1/2 signaling [22]. Downregulation of antigen presentation may also be attributed to epigenetic changes, which may be overcome through the use of DNA methylation inhibitors and histone deacetylase inhibitors [23]. Given the role of the gut microbiome in regulating the mucosal immune system, it has also been shown to influence the response to immune checkpoint inhibitors [24]. Consequently, modulating the gut microbiome, for example, through fecal transplantation or simply by encouraging high-fiber diets in patients, may further increase the efficacy of immune checkpoint inhibitors [25]. Interestingly, however, recent evidence suggests that taking dietary supplements such as probiotics may hinder the response to checkpoint inhibitors by lowering the diversity of the gut microbiome, warranting further research in the area [25]. A summary of the various combinatory immunotherapies discussed and their corresponding synergistic effects on the immune system is provided in Figure 2. 

Adoptive cell transfer and vaccines

Adoptive cell transfer (ACT) is another approach used in immunotherapy, where the patient’s own T lymphocytes with anti-tumor activity are identified, expanded in vitro and re-infused into the patient, often along with growth factors. CAR-T cell therapy is a particularly attractive strategy, where the patient’s T cells are genetically engineered to express modified CARs that target surface antigens whose epitope is unique to cancer cells. This approach is also attractive in that CARs are HLA-independent, eliminating the need for haplotype matching. However, while CAR-T therapy has had success in the treatment of hematological tumors, their application in solid tumors has been largely limited [26]. This can firstly be attributed to the lack of specific targetable antigens. For example, while CD19 has proven to be the most successful target antigen for CAR-T cell therapy due to its ubiquitous expression in almost all B cell malignancies, it is also expressed by non-malignant B cells [27]. However, fortunately, the B cell aplasia and hypogammaglobulinemia associated with this lack of specificity can be easily managed. Further barriers to CAR-T cell therapy include problems in T cell homing, infiltration, and subsequent survival in the tumor microenvironment. Infiltration may be improved through the engineering of CAR-T cells capable of degrading cellular components, such as αvβ6 integrin, VEGF receptor 2 and heparan sulphate proteoglycans (through heparanase release) [28-30]. 

To counteract the hostile and immunosuppressive nature of the tumor microenvironment, and hence improve T cell survival, several strategies can be employed. Such strategies include engineering CAR-T cells that 1) are resistant to TGF-β suppression via dominant-negative TGF-β receptor expression, 2) can counteract the action of reactive oxygen species (ROS) through catalase expression, reducing H_2_O_2_, and 3) can convert IL-4’s suppressive effects to a stimulatory one through engineering chimeric receptors that express the IL-4 receptor ectodomain [31-33]. CAR-T cell therapy may also provide a selective pressure, facilitating the emergence of antigen loss variants with time [26,34]. This can be overcome by enabling the CAR-T cells to target more than one antigen. For instance, through engineering the extracellular portion of the CD16-chimeric receptor to express an FcγR domain, it is able to bind to any therapeutic antibody directed against any tumor-associated antigen, triggering both a cellular immune response and antibody-dependent cellular cytotoxicity [35]. Alternatively, the two-component SUPRA CAR system has recently been developed, which is composed of a receptor expressed on T cells (zipCAR) and an antigen-binding component (zipFv). In this system zipCAR expressing T cells are activated once a zipFv component containing a matching leucine zipper is added. SUPRA CARs are universal in that multiple zipFv components expressing the same leucine zipper but different antigen-binding domains can be added, allowing the targeting of multiple antigens. Thus, throughout the course of therapy, antigen specificity can be altered depending on the patient response to improve treatment efficacy [36]. 

A further strategy is the dual recognition of tumor-associated antigens expressed by the same cell by two CARs [37,38]. As well as enhancing T cell activation, this approach may also be able to reduce on-target/off-tumor toxicity by improving specificity to the target tumor, protecting normal tissues. Alternatively, toxicity can be reduced by combining CARs directed against target antigens with inhibitory CARs (iCARS). The idea is that target antigens for CAR-T cell therapy are also expressed by healthy tissues, albeit at lower levels compared to the tumor. iCARS are able to produce inhibitory signals that can override this low level of T cell activation against target antigens expressed by healthy cells, whilst enabling CAR-induced T cell activation against tumor cells [37]. For example, the inhibitory receptors CTLA-4 and PD-1 can be used in iCARs to negatively regulate the activation of T lymphocytes against normal tissue, thus reducing off-target toxicity [39]. Toxicity associated with CAR-T cells may also relate to the use of retroviral (RV) and lentiviral (LV) vectors, which may trigger immune and inflammatory responses [40]. Instead, alternative systems, such as the transposons *piggyBac* (PB) and *Sleeping Beauty* (SB) can be utilized, which also simplify and reduce the costs associated with transduction [26]. Furthermore, as they do not utilize reverse transcription, the likelihood of aberrant gene rearrangements is minimized. 

Most targeted antigens in immunotherapy are not tumor-selective and rather are just overexpressed in tumors [26]. However, neoantigens are not encoded by the normal genome and instead arise in tumors as a result of driver mutations and as by-products of increasing genetic instability (passenger mutations), often rendering the pattern of expression as highly unique to the individual [41]. Whilst this generally means neoantigens are not practical for CAR-T therapy, distinct neoepitopes have been identified. For example, MUC-1 targeting CAR-T cells have been shown to significantly delay tumor progression [42]. Furthermore, neoantigen-directed T cells from a patient or donor can also be identified and expanded in vitro for treatment, or alternatively T-cells can be genetically engineered to express neoantigen-specific TCRs [43-44]. Enhancement of antigen presentation through stimulation of the innate immune response and dendritic cell function, for instance by using type I IFN and toll-like receptor (TLR) ligands, may also promote the formation and presentation of neoantigens [45]. In turn, this may mount a more significant response by neoantigen-directed T cells. These methods combined overcome the problem of a lack of suitable neoantigens and alterations in antigen processing and/or presentation, which has been associated with impaired anti-tumor activity [46].

Vaccine based approaches targeting neoepitopes can also be utilized, typically employing synthetic peptides, DNA or RNA to encode the neoantigen. Whilst some neoantigens are shared between various tumors and patients, the repertoire is rather small [47]. Consequently, this largely limits the use of neoantigen-based vaccines for the treatment of cancer. However, with the advent of next generation sequencing technologies, mutations specific to an individual patient’s tumor can be identified, leading to the development of tailored neoantigen-based vaccines. Such vaccines exert their effect through numerous mechanisms, including priming the immune system and enhancing the response by CTLs [20,48]. Furthermore, poly-neoantigen vaccines can be utilized to facilitate an augmented response. They may also be used to help overcome the issue of tumor heterogeneity and minimize the risk of clonal expansion of antigen loss variants of tumor cells, which can confer treatment resistance [47]. Through this effect, neoantigen-based vaccines are able to act as a crucial adjunct for both ACT therapy and treatment with immune checkpoint inhibitors [12,20].

Challenges and future directions

To facilitate the widespread adoption of immunotherapy for the treatment of cancer, a few barriers must be overcome first. Most importantly, toxicity resulting from enhanced activation of the immune system is an obstacle that prevents the regular use of immunotherapy, particularly for combinatory regimens. Such immune-related adverse events (irAEs) include acute episodes of autoimmune-like disease, as seen with immune checkpoint inhibitors, making efficacy and safety studies essential when considering such therapies. Furthermore, given the inherent complexity of tumors, preclinical models that accurately reflect the natural course of tumor development and the associated immunosuppressive microenvironment must be utilized, such as genetically engineered mouse models. Consideration also needs to be given to the route of delivery and other pharmacokinetic properties of immunotherapies in order to maximize bioavailability to the target tumor site, for example through the use of nanoparticle drug delivery systems. In addition, immunotherapy is often limited by the use of conventional chemotherapy as first-line treatment. As a result, by the time, immunotherapy is utilized the patient’s immune system may already be compromised due to advanced disease and/or previous therapy. It is therefore essential that appropriate treatment regimens that optimize the dose, schedule and duration of therapy are designed. 

Given the abundance of potential target molecules and the wide array of combinatory therapies, it is imperative that biomarkers are developed to help predict tumor responses. This will enable immunotherapy to be tailored to the individual patient, improving efficacy, and reducing toxicity. For instance, in addition to CAR-T cell therapy, neoantigens may be utilized as predictive biomarkers to identify tumors more amenable to checkpoint inhibitor therapy, due to the correlation between the number of mutations/neoantigens and the therapeutic response [49]. Similarly, tumor mutational burden and the presence of certain immune inhibitory molecules such as PD-L1, CTLA-4, and IDO1 can be used to predict response to checkpoint inhibitor blockade in a range of cancers, including NSCLC, renal cell carcinoma, bladder cancer, and melanoma [50]. To facilitate a shift to an era of more personalized cancer therapy, cost-effective and practical methods for identifying relevant biomarkers and/neoepitopes, and categorizing cancers according to the underlying immunosuppressive mechanism, must be developed. 

## Conclusions

While immunotherapy is still very much in its infancy, it has already shown huge promise and is well-aligned in becoming the mainstay treatment for cancer. In particular, the use of combination therapies and strategies to boost the immune response appear to be particularly attractive approaches, although careful consideration must be given to minimize the likelihood of irAEs. Tailoring immunotherapy to the individual patient through the use of neoantigens and predictive biomarkers should also be further explored in order to improve treatment efficacy, whilst minimizing toxicity. Given the heterogeneous nature of tumors, it is crucial that these different strategies are explored simultaneously and synergistically to ensure immunotherapy lives up to its potential in improving the treatment for the majority of cancers. 
